# Weight management during pregnancy: a qualitative thematic analysis on knowledge, perceptions and experiences of overweight and obese women in Singapore

**DOI:** 10.1080/16549716.2018.1499199

**Published:** 2018-08-06

**Authors:** Alvona Zi Hui Loh, Kellynn Qi Xuan Oen, Ischelle Jing Yuan Koo, Ying Woo Ng, Jason Chin Huat Yap

**Affiliations:** a Yong Loo Lin School of Medicine, National University of Singapore, Singapore, Singapore; b Department of Obstetrics and Gynaecology, National University Hospital, Singapore, Singapore; c Saw Swee Hock School of Public Health, National University of Singapore, Singapore, Singapore

**Keywords:** Global health, public health, maternal health, nutrition, overweight, obesity, beliefs, behavior, qualitative methods, Asia

## Abstract

**Background**: The effect of maternal weight on pregnancy and birth outcomes is of great public health concern. For example, overweight and obesity among pregnant women is associated with adverse reproductive health outcomes including gestational diabetes, birth defects, macrosomia and infant mortality.

**Objectives**: To understand the knowledge, perceptions and experiences during pregnancy among overweight and obese women in Singapore.

**Methods**: This is a cross-sectional study which employed qualitative techniques. The inclusion criteria is as follows: (1) Women who were overweight or obese before or during first trimester of pregnancy, (2) are able to report their specific pre-pregnancy or first trimester Body Mass Index (BMI), or weight and height, (3) had singleton pregnancy when overweight, are post-delivery for the pregnancy in which they were overweight, and (4) are 21 to 55 years old. Fifteen interviews were voice-recorded and transcribed verbatim. Then, two researchers independently performed the thematic analysis of the transcripts.

**Results**: The findings revealed that women had gaps in knowledge on the adverse effects of maternal overweight and obesity during pregnancy, and various misconceptions on diet and weight management during pregnancy were prevalent. Conflicting data was obtained for perceptions on the importance, difficulty and levels of motivation for weight management during pregnancy.

**Conclusion**: There is a pressing need to address knowledge gaps and misconceptions of pregnant women who are overweight or obese. The importance of weight management during pregnancy should be emphasized, and enabling factors put in place.

## Manuscript

### Background

The effect of maternal weight on pregnancy and birth outcomes is of great public health concern [1]. Both developed and developing countries are observing a rapid increase in the prevalence of obesity [2–4]. Overweight and obesity among pregnant women is associated with numerous adverse reproductive health outcomes [5–25]. These adverse outcomes include increased risk of gestational diabetes [5], pregnancy-induced hypertension and pre-eclampsia [6], birth defects [7], large for gestational age (LGA) or macrosomia (> 4500 g) which may lead to possible birth injury [8], shoulder dystocia [9], delivery through caesarean section [10–12], prolonged labor process [13], severe asphyxia-related outcomes in term infants [14], postpartum anemia [15–17] and postpartum weight retention [18–22]. Serious implications on the fetus include increased risks of infant mortality due to increased mortality risk in term births and an increased prevalence of preterm births [23,24]. Furthermore, the Confidential Enquiry into Maternal and Child Health (CEMACH) [25] found that obesity could be a contributor to maternal and fetal mortality as an outcome.

Most of the aforementioned studies [5–25] followed maternal overweight and obesity definition as Body Mass Index (BMI) above the normal range of 19.8 to 26.1 (Institute of Medicine, 1990). This definition lasted until 2009, when the Institute of Medicine (IOM) published revised pregnancy weight gain guidelines based on prepregnancy BMI ranges recommended by the World Health Organization [26]. Thereafter, normal weight is defined as a BMI of 18.5–24.9, overweight as a BMI of 25–29.9, and obesity as a BMI of 30 or greater. These ranges are independent of age, parity, smoking history, race, and ethnic background.

In view of the aforementioned adverse effects, Western studies [27–33] from Australia, the USA, the UK have explored the perceptions of women during pregnancy, which targeted women who were White, Latina and African-American. For example, in a study [27] which compared perceptions of White women and African American women, it was found that African American women were more concerned with inadequate weight gain whereas White women more frequently expressed worry on excessive weight gain, and that the primary cited barrier of a healthy diet was the high cost of fresh produce. In another Australian study, the authors reported that conversations on weight and gestational weight gain can be conflicting, confusing and judgmental, and that healthcare providers should approach the issue of maternal obesity in an informative but non-judgmental way [28], while a study in Boston targeting Latina women [29] revealed that in comparison to healthy weight and obese women, overweight women least often reported viewing weight as important, making efforts to control their gestational weight gain, being aware of the role of diet on gestational weight gain, and receiving gestational weight gain advice from health-care providers.

To date, no data on women’s knowledge, perceptions and experiences of overweight and obesity during pregnancy in an Asian population is available. Given that socio-cultural differences between the Asian and Western populations exist, as well as across the different ethnicities, the knowledge, perceptions and experiences of overweight and obese women during pregnancy in Asian communities is likely to differ from that of the West. Singapore is an Asian country that is multi-ethnic, multi-cultural and multi-lingual, with major ethnic groups consisting of the Chinese, Malays, Indians, as well as others such as Eurasians. Given this cultural diversity in the population, health belief models and attitudes toward health among women in this country is likely to differ from that of the West.

The objectives of our study are as follows: (1) to explore the understanding and knowledge of the effects of maternal overweight and obesity during pregnancy on the outcomes for the mother and child, (2) to investigate the perceptions on the importance of weight management during pregnancy for overweight and obese women, and (3) to explore the experiences on weight management during pregnancy among women who were overweight or obese before or during first trimester of pregnancy, in order to develop clinical pathways and shape policies to improve the way(s) in which overweight and obese women manage their weight during pregnancy.

## Methods

### Study design

We conducted a qualitative study via a phenomenological approach [34] on women who were overweight or obese before or during first trimester of pregnancy. First trimester BMI was used as an estimate for pre-pregnancy BMI, if the participant had her height and weight measurements only during first trimester and not before pregnancy. The approach we adopted studies the empirical world from the perspectives of the subject, not the researcher. This implies that we aimed to understand the knowledge, perceptions and experiences on weight management during pregnancy from the angles of the recruited subjects, i.e. the women who were overweight or obese before or during first trimester of pregnancy. A set of study-specific topics (Table 1), i.e. the interview guide, derived from our literature review to answer our research question, was developed by AZHL, YWN and JCHY to explore women’s experiences during pregnancy in three domains: (1) Knowledge on maternal overweight and obesity during pregnancy, (2) perceptions on weight management during pregnancy, (3) experiences on weight management during pregnancy. The literature review was conducted by AZHL, JCHY and YWN.

**Table 1.** Qualitative interview guide.

General demographics	What is your age, race and occupation? Are you currently married? How many years have you been married? How many children do you have? How old are they? At which hospital did you give birth to your children? Have you had any miscarriages, abortions or stillbirths? Do you have any underlying or past medical conditions? Have you had any past surgeries? Do you have any family history of medical conditions? Where do you live? Who do you live with? Did you or do you require financial assistance? What is your highest educational level? Who takes care of you during pregnancy? Who takes care of your children now? Did you attend appointments with O&G clinic according to schedule?
Information on previous pregnancies	(15) Were your children born full term, premature or post term? How many weeks were they at birth? (16) What were the birth weights (in kg) of your children? (17) Were your children born by normal vaginal delivery, assisted vaginal delivery (e.g. forceps or ventouse) or Caesarian section? Why did you need it? Please elaborate. (18) Were there complications during your pregnancy or delivery? (19) Were your children well after delivery?
Weight-related information	(20) What was your BMI (or height and weight) before pregnancy or during first trimester of pregnancy? (21) What is your current height and weight? (22) Is this your usual height and weight? (If not, what is your usual height and weight?) (23) How much weight did you gain during pregnancy, at your heaviest? (If > 1 pregnancy, state the pregnancy in which the most weight was gained). (24) Is anyone in your family overweight or obese?
Information on lifestyle	(25) Did you try to reduce your weight before pregnancy? Why? (26) Did you try to reduce your weight during pregnancy? Why? (27) What kind of diet did you have when pregnant, e.g. in terms of portion and type of food? (28) How much food do you think a pregnant lady should eat during pregnancy? Why? (29) Did you change your diet after you were pregnant, e.g. in terms of portion and type of food? Why? (30) Did you exercise when you were pregnant? If so, what kind of exercise did you do, and how frequently?
Knowledge on Maternal Overweight and Obesity during Pregnancy	(31) What do you know about overweight and obesity during pregnancy? (32) Can you name some problems to the mother and child, if the mother is overweight or obese during pregnancy? (33) (If could answer above question) How did you know about these problems? (34) Did anyone in tell you how the mother’s overweight and obesity can affect the outcomes of pregnancy?
Perceptions on Weight Management during Pregnancy	(35) Do you think managing weight during pregnancy, for an overweight or obese lady, is difficult? Why? (36) Did you think that weight management during pregnancy, for an overweight or obese lady, is important? (37) Did you feel motivated to manage your weight during pregnancy? Why?
Experiences on Weight Management during Pregnancy	(38) Have you tried ways to manage your weight during pregnancy? (39) Have you looked for or participated in programs or support groups, to help you in terms of weight management during pregnancy? (40) How do you think women can manage their weight safely and effectively during pregnancy?

### Study setting

Fifteen individual in-depth one-to-one interviews were conducted from 16 May 2016 to 31 May 2016 on the premises National University Hospital System (NUHS) or National University of Singapore (NUS). The study was conducted in Kent Ridge, Singapore. The region is a neighborhood located in Pasir Panjang, in the Queenstown Planning Area of Singapore which covers an area of approximately 20.43km^2^. One-to-one interviews took place in various unoccupied rooms, such as an unoccupied clinic consultation room or university tutorial room, where there was space for the interviews to be conducted in privacy.

### Participant recruitment

The inclusion criteria is as follows: (1) Women who were overweight or obese before or during first trimester of pregnancy, (2) are able to report their specific pre-pregnancy or first trimester Body Mass Index (BMI), or weight and height, (3) had singleton pregnancy when overweight, (4) are post-delivery for the pregnancy in which they were overweight, to ensure that they have undergone the full spectrum of pregnancy and (5) are 21 to 55 years old. We used first trimester BMI as an estimate for pre-pregnancy BMI, if the participant has her height and weight metrics only for first trimester visit but not before pregnancy. The exclusion criterion of participants is as follows: women who fulfill the inclusion criteria, but are unwilling to participate in our study.

The minimum age of 21 was determined by Singapore’s age of majority, and the maximum age of 55 was determined by the oldest age at which most women would have reached menopause. We did not have any inclusion criteria aiming at different educational level among the informants, and were open to participants of all educational levels if they indicated interest and were eligible for the study.

The authors designed recruitment posters stating the study aim and participant eligibility criteria, which were written in the four major languages used in Singapore: English, Chinese, Malay and Tamil. The corporate communications department of National University of Health System (NUHS) and National University of Singapore (NUS), on behalf of the investigators of this study, placed recruitment posters at the general notice boards of NUHS and NUS, to invite women who fulfill the eligibility criteria to participate in our study, if they are interested and willing to. We also placed soft copies of recruitment posters at online platforms, such as the events website of NUS and social media platform Facebook, for access by members of the public. Eligible women who were interested and willing to participate responded by contacting the first author (AZHL) of this study. Then, interviews were arranged for and conducted with the participants.

### Data collection

All interviews were conducted face-to-face by two of the researchers (AZHL, KQXO). For example, to assess the knowledge of the participants on overweight and obesity during pregnancy, we asked, ‘What do you know about overweight and obesity during pregnancy?’ All participants reported that they were fluent in English and required no translation. Each interview was held in a comfortable area, lasting for approximately 30 minutes. The inclusion criteria were described to the participants before each interview, to ensure that they were eligible to participate. A free-flow discussion was permitted during the interviews, although themes in the interview guide derived from our literature review to answer our research question, were used to help facilitate the process. The interviewers were open to free discussion and the interview guide only served to prompt the participants on possible areas to explore. All 15 participants who signed up for the study were interviewed. Saturation was reached when the members of the research team found that new data tended to be redundant of data already collected. For example, in interviews, when we began to hear the same comments again and again, data saturation is being reached [35].

### Data preparation

All digital audio voice recordings made during the interviews were transcribed verbatim by two of the researchers (AZHL, KQXO). The responses were categorized into themes and sub-themes using context analysis. Then, two researchers (AZHL, KQXO) independently analyzed the transcripts.

### Data analysis

Two members of the research team (AZHL, KQXO) independently reviewed and ratified the themes that surfaced. Thematic analysis was done with a focus on identifying and describing both implicit and explicit ideas within the data (i.e. themes) that were derived from the interviews with our subjects, after interviews were transcribed verbatim in a way that the transcript retained the information needed from the verbal account, and in a way which is true to its original nature.

Specifically, the investigators followed the following six steps of thematic analysis [36]:

1. Both investigators immersed themselves in the data to the extent that both were familiar with the depth and breadth of the content, through repeated reading of the data, and actively searching for meanings, patterns etc. Both investigators read through the data at least twice before the coding phase.
2. An initial list of ideas about what is in the data and what is interesting about them was generated. Codes which identify a feature of the data (such as semantic content or latent) that appears interesting to the investigators were noted, for example by writing notes on the text during analysis, and using highlighters and colored pens to indicate potential patterns. Coding was done manually, with the investigators working systematically through the entire data set. Each data item was given full and equal attention, and the aspects worthy of note in the data items that may form the basis of repeated patterns (themes) were identified. Identified codes were then matched with data extracts that demonstrate that code, with all actual data extracts coded and collated together within each code. This involved copying extracts of data from individual transcripts and collating each code together using file cards.
3. Next, thematic search began after all data was coded and collated, and a long list of different codes identified across the data set was generated. The investigators analyzed the codes and looked into how different codes may combine to form an overarching theme, with the use of visual representations including mindmaps and tables. Some initial codes formed main themes, whereas others formed sub-themes.
4. Thereafter, the investigators reviewed the themes, by refining the themes such as combining themes that have the same core meaning, breaking down themes with different core meanings, and discarding themes that have insufficient evidence to support them. All coded data extracts for each theme were read and given consideration on whether they appear to form a coherent pattern. Also, consideration was given on the validity of individual themes in relation to the data set, and whether the thematic map accurately reflects the meanings evident in the overall data set. The investigators re-read the entire data set to ascertain whether the themes ‘work’ in relation to the data set, and to code additional data within themes that were missed in earlier coding stages.
5. Subsequently, the investigators defined and named the themes, through identifying the essence of what each theme is about, and what the themes overall are about, on top of determining what aspect of the data each theme captures. For each individual theme, a detailed analysis was conducted and written. The investigators identified the ‘story’ each theme reflects, and considered how the themes fit into the broader overall “story that is being told through the data in relation to the research question. In the refinement phase, the investigators identified whether or not a themes contains sub-themes which are themes within a theme that can be useful for giving structure to a particularly large and complex theme, and for demonstrating the hierarchy of meaning within the data. The themes were defined clearly, and were tested on their clarity based on whether the scope and content of each theme could be described in a few sentences.
6. Last, with a set of fully worked-out themes, the final analysis and write-up was done to tell the complicated story of the data in a way which convinces the reader of the merit and validity of the analysis. A concise, coherent, logical, non-repetitive, and interesting account of the story which the data tells was written by the investigators. Member checking was performed by IJYK who validated the themes which emerged from the analyses.

### Ethics, consent and permissions

Ethics approval was obtained from the National University of Singapore (NUS) Institutional Review Board. The IRB approval number is NUS-2981 (NUS-IRB Ref Code: B-16–100). Written informed consent to participate in the research study was obtained from all women who signed up for the study. The aim of the study was clearly explained to each participant and they were informed of the anonymity and confidentiality of the responses. Participants were also informed that their participation was voluntary and they could withdraw from the session at any time.

## Results

The themes identified were: (1) Levels of knowledge on the implications of maternal overweight and obesity, (2) Misconceptions on the implications of maternal overweight and obesity during pregnancy, as well as perceptions on the (3) Difficulty of weight management during pregnancy, (4) Importance of weight management during pregnancy, (5) Motivation of weight management during pregnancy, (6) Experiences on weight management during pregnancy. Sub-themes for each main theme were also identified. These themes were relevant in answering our research question, besides being of significant use in allowing the investigators achieve the objective of the study that is to develop clinical pathways and shape policies to improve the way(s) in which overweight and obese women manage their weight during pregnancy.

The results will describe the themes extracted from the transcripts, such as misconceptions on the implications of maternal overweight and obesity during pregnancy, and then the sub-themes such as a need to consume twice the usual portion of food during pregnancy and hunger from the woman is due to hunger from the baby. Representative quotation(s) from the transcripts to illustrate the sub-theme will be included in the paper. Subsequently, the syntheses from the authors based on the transcripts, will be presented. In this section, only lines in quotations are informants’ stories as no quotations have been paraphrased. All other aspects presented in the paper are authors’ discussions and syntheses based on the quotations.

### General demographics of participants

Fifteen in-depth interviews were conducted for the 15 women who participated in the study. In this paper, participants are numbered from ‘Participant 1’ to ‘Participant 15’, and referred to as ‘P1’ to ‘P15’ for short. Participants’ ethnicity varied, with women of Chinese, Malay or Indian ethnicity. All participants had at least secondary school education, and two participants (P7, P8) had nursing knowledge as they were working as nurses in a hospital. The rest of the participants were from the teaching, service, or sales industry, or unemployed, with no medical or nursing knowledge. Demographics of the participants, such as information on their age, ethnicity, occupation, type of work, marital status, number of years married, number of children and other information on pregnancy history and weight, are presented in Table 2.

**Table 2.** General demographics of participants, N = 15.

Characteristic	Median (IQR)
Age (years)	40 (37 – 48)
Pre-pregnancy or first trimester BMI (kg/m^2^)	27.8 (25.5 – 30.8)
Highest weight gain during pregnancy (kg)	12 (10 – 20)
Number of years married	12 (10 – 18)
Number of children	3 (2 – 3)
**Characteristic**	**N (%)**
Race	Chinese: 6 (40.0) Malay: 6 (40.0) Indian: 3 (20.0)
Occupation	Receptionist work, administrative work, cashier services: 7 (46.7) Childhood educator: 2 (13.3) Nurse (with nursing knowledge): 2 (13.3) Sales assistant: 1 (6.67) Management assistant: 1 (6.67) Housewife: 1 (6.67) Unemployed: 1 (6.67)
Has medical or nursing knowledge	No: 13 (86.7) Yes (nursing knowledge): 2 (13.3)
Highest education level	Secondary education: 8 (53.3) Pre-University: 2 (13.3) University: 5 (33.3)
Marital status	Married: 13 (86.7) Divorced: 1 (6.67) Widowed: 1 (6.67)

Some of the participants also experienced the problems known to be associated with maternal overweight and obesity, such as stillbirth (P1, P4), atrial septal defect in the child (P2), macrosomia (P2, P3, P4, P12), and maternal gestational diabetes (P10, P11, P12). Other problems reported during pregnancy were abruptio placentae (P1) and threatened abortion (P2), premature rupture of membranes (P14) and hyperemesis gravidarum (P15).

### Levels of knowledge on the implications of maternal overweight and obesity (Table 3)

Four main levels of knowledge were demonstrated by the women who participated in the interviews. These were: (1) No knowledge on any of the problems of maternal overweight and obesity, (2) erroneous knowledge on the problems of maternal overweight and obesity, exemplified by citing examples such as conditions in the child not associated with maternal overweight or obesity, (3) limited knowledge on the problems of maternal overweight and obesity, exemplified by stating that it is generally not good, (4) some knowledge on the problems of maternal overweight and obesity, exemplified by stating that it is generally not good, with elaboration using maximum two correct and specific examples on implications to the woman or child.

**Table 3.** Levels of knowledge on the implications of maternal overweight and obesity on pregnancy and birth outcomes.

Levels of knowledge	Sub-themes
**Level 1**: **No knowledge on any of the problems of maternal overweight and obesity**	Unable to name any implications of maternal overweight and obesity on pregnancy and birth outcomes
**Level 2**: **Erroneous knowledge on the problems of maternal overweight and obesity**	(2) Able to specify that maternal overweight and obesity is generally not good (3) However, the problems cited were irrelevant to overweight and obesity during pregnancy, such as asthma and sinusitis in the child
**Level 3**: **Limited knowledge on the problems of maternal overweight and obesity, exemplified by stating that it is generally not good**	(4) Able to specify that maternal overweight and obesity may have poor outcomes, but unsure of what these poor outcomes were
**Level 4**: **Correct and specific examples on implications to the mother and child**	(5) Able to specify that maternal overweight and obesity is generally not good (6) Able to cite specific outcomes, which includes maximum of 2 outcomes from the following list: Difficulty in natural vaginal delivery, maternal gestational diabetes, diabetes in the child, maternal hypertension, macrosomia or large for gestational age (LGA) in the baby (7) Only one participant (P6) could name fetal demise as a possible outcome (8) None stated stillbirth or congenital malformations in the baby as possible outcomes

***Level 1: No knowledge on the effects of maternal overweight and obesity***

A gap in knowledge on the effect of maternal overweight and obesity on pregnancy and birth outcomes was demonstrated in some interviews.

“Actually, all the while I have no idea on problems of overweight and obesity during pregnancy, but I know that I am obese.” (P9)

***Level 2: Erroneous knowledge on the effects of maternal overweight and obesity***

Some participants stated that maternal overweight and obesity is associated with poorer outcomes on the child. However, the problems cited were erroneous and irrelevant to maternal overweight and obesity, such as asthma, sinusitis and eczema in the child, which are more likely to be attributed to genetic inheritance of atopy instead of maternal overweight and obesity.

“Overweight and obesity is not good for the mother during pregnancy and after giving birth there could be a lot of problems. The child may be affected as well. For example, my child had asthma, sinusitis and eczema.” (P5)

When probed about how this knowledge was obtained, participants often stated that they made the conclusion based on their own pregnancy experiences.

“I knew through my experience with my own child.” (P5)

Therefore, the participant’s knowledge can be said to be gained through her own observation of her child, which could be done only after her pregnancy. However, this participant lacked accurate knowledge on the outcomes associated with maternal overweight and obesity before the delivery of her child, and was unaware that her observations were in fact inaccurately attributed to maternal overweight and obesity.

***Level 3: Limited knowledge on the effects of maternal overweight and obesity, that it is generally not good***

Several participants were able to state that maternal overweight and obesity is generally not good, although they were unsure of what these poor outcomes were.

‘Obese mothers tend to give birth to unhealthy kids.’ (P2)

***Level 4: Some knowledge on the problems of maternal overweight and obesity, exemplified by stating that it is generally not good, with elaboration using correct and specific examples on implications to the woman and child***

Several participants demonstrated knowledge on some problems of maternal overweight and obesity, and supported this with correct and specific examples on implications to the woman and child. Commonly cited examples from the participants were: difficulty in natural vaginal delivery, maternal hypertension, maternal gestational diabetes, diabetes in the child, macrosomia or large for gestational age (LGA) in the baby.

‘I only know that there could be difficulty in natural vaginal birth for overweight or obese women.’ (P1)

‘There could be diabetes and hypertension to the mother, but that is all that I know.’ (P4)

However, many participants could only state up to two correct examples of the implications of maternal overweight and obesity on the woman and child, which demonstrates a lack of awareness of potential issues to expect in the pregnancy when they were overweight.

### Misconceptions on the implications of maternal overweight and obesity during pregnancy (Table 4)

From our findings, four major misconceptions were identified among our study population, which have the potential to modify pregnant women’s decisions on diet and exercise. The extent of influence of these misconceptions on the pregnant women’s lifestyle is unknown, but each has the potential to further exacerbate the problem of maternal overweight and obesity during pregnancy.

**Table 4.** Misconceptions on the implications of maternal overweight and obesity during pregnancy.

Misconception	Sub-themes
**A need to consume twice the usual portion of food during pregnancy**	Misconception: There is a need for consumption of twice the usual portion of food as pregnant women should eat for two people, the mother herself and the baby, during the pregnancy Although cited as a Chinese belief from some of the participants, women of other ethnicities have also discussed this misconception This may lead to excessive food consumption by women during pregnancy if they believe that they need to consume twice the usual amount of food in their diet
**Insufficient food consumption by the mother during pregnancy will have effects on the baby after delivery, such as the baby drooling**	(4) Misconception: The cause of baby drooling is attributed to insufficient food consumption by the mother when she was pregnant (5) This belief may cause pregnant women to eat more during pregnancy to avoid speculations that she did not eat enough when she was carrying the baby, which may lead to the baby drooling after the delivery
**Hunger from the mother is due to hunger from the baby**	(6) Misconception: Hunger experienced by the pregnant lady signifies hunger from the fetus (7) This belief may motivate women to eat every time they feel hungry, as ignoring their hunger may be interpreted as neglect of their baby’s hunger
**Women should not exercise during pregnancy, as it can injure the fetus or lead to miscarriage**	(8) Misconception: Women should not exercise during pregnancy as it can lead to fetal injury and miscarriage (9) This view on exercise during pregnancy runs across all ethnicities and is a commonly cited reason for not exercising during pregnancy (10) This belief may cause the mother to worry about potential harm to the fetus from exercising during pregnancy

### A need to consume twice the usual portion of food during pregnancy

The belief that a pregnant lady needs to consume twice the usual portion of food for herself and on behalf of the baby was demonstrated in several interviews. This misconception was sometimes attributed to the Chinese’ view on the portion of food intake needed during pregnancy.

“The Chinese believe that pregnant women need to eat double the number of meals for the baby.” (P1)

“As I’m Chinese, I think most Chinese have this thinking that pregnant ladies should consume double portion.” (P8)

However, this belief was also displayed in other women who were not of Chinese ethnicity, e.g. of Malay ethnicity.

“People always say, ‘eat for two, eat for two’ when pregnant.” (P2)

This misconception may lead to excessive food consumption by women during their pregnancy, if they are misled to believe that they need to consume twice the usual amount of food in their diet. This has implications on both the woman and child. For example, excessive food intake could cause excessive weight gain by the woman, which can translate to increased fetal weight and hence a larger baby, which poses problems during delivery.

### Insufficient food consumption by the woman during pregnancy will have effects on the baby after delivery, such as the baby drooling

There is also the belief that insufficient food consumption by the woman during pregnancy translates to effects on the baby, such as the baby drooling.

‘Some old wives’ tale says that if you feel like eating something but don’t eat, then your baby will drool when born…. It is a known old wives tale. Everybody talks about it. When they see a baby drool, the first thing that comes into mind is, it must be that the mother didn’t eat something during pregnancy.’ (P2)

If old wives tales such as the example cited above is prevalent in the community, women may be inclined to eat each time she has a craving, in order to avoid effects such as drooling by the baby after delivery. The pressure to satisfy her cravings may be exacerbated if such views are shared by other members of the family, such as the husband or mother-in-law, who may put the blame on the woman if the baby is observed to drool after birth.

### Hunger from the woman is due to hunger from the baby

Another belief identified during the interviews was the perception that hunger experienced by the pregnant lady signifies hunger from the fetus.

‘If the pregnant lady is hungry, it could mean that the baby is hungry, so not eating will affect the baby.’ (P1)

The perceived link between hunger experienced by the pregnant woman during pregnancy and the baby’s hunger may lead to women eating whenever they are hungry, for fear that the baby will feel hungry if food is not consumed immediately.

### Women should not exercise during pregnancy, as it can injure the fetus or lead to miscarriage

Concerns on exercise during pregnancy were identified in our study population. Common fears on the effects of exercise during pregnancy included fetal injury and miscarriage, and even preterm labor in the third trimester.

‘I did not exercise at all during pregnancy. I didn’t know what exercise to do, and was afraid that it would cause a miscarriage.’ (P1)

‘I did not exercise when I was pregnant. I was scared that it would injure the fetus.’ (P3)

‘Some people say that if you exercise a lot, there would be miscarriage, or you may affect the child, so we try to be safe and not exert ourselves. The mother would be worried of these things.’ (P10)

For a participant who did deliberate exercise, there were also concerns on exercising during the first and third trimester, which were deemed to be dangerous periods during which the woman exercising could potentially harm the baby.

‘I go a few times for swimming, then after that I had slow walks. For swimming, I went there for 30 minutes to one hour, as my house is near the swimming pool. I went about once a month, during my second trimester. I did not swim in the first or third trimester. For first trimester, we don’t usually exercise as we need to be careful, so I only went in second trimester. In third trimester, my mother-in-law didn’t allow me to go.’ (P9)

A participant whose husband is a fitness trainer, reported that she was confident to exercise in all three trimesters of pregnancy, as her husband had the knowledge of appropriate exercises for pregnant women throughout pregnancy.

‘I wasn’t afraid that exercise may affect my pregnancy, as my husband is a fitness trainer, so he had a lot of exercises suitable for pregnant women so I was quite lucky.’ (P15)

However, she reported that without her husband’s expertise, she would have been wary of exercising when pregnant, especially the first and third trimester. She would not have done exercises such as weights and jogging.

‘If not for my husband, I would have been more cautious, I wouldn’t have thought of exercising during first and last trimester, and I wouldn’t do weights and wouldn’t feel doing jogging. I wouldn’t even think of exercising.’ (P15)

She also cited various reasons that some people may avoid exercise during certain periods, such as ‘fragility’ and risk of miscarriage in the first trimester, and preterm labor in the third trimester.

‘Some people think that in first trimester, you are more fragile, and may easily get miscarriage especially if high impact exercise…. For third trimester, people are afraid of early labor, like when you are brisk walking you may feel like giving birth.’ (P15)

### Perceptions on difficulty of weight management during pregnancy (Table 5)

Three broad perceptions on the levels of difficulty on weight management during pregnancy were sieved from the participants’ responses: (1) Weight management during pregnancy is difficult, (2) Weight management during pregnancy is not difficult, (3) The difficulty of weight management during pregnancy depends on the individual. Of these, the perception that managing weight during pregnancy is difficult was reported by many of the participants, and attributed this view to a multitude of reasons.

**Table 5.** Perceptions on difficulty of weight management during pregnancy.

Perception	Sub-themes
**Weight management during pregnancy is difficult**	Difficult due to worry of affecting the baby during weight management Difficult due to ‘pampering’ of the expectant mother during pregnant Difficult due to cravings during pregnancy
**Weight management during pregnancy is not difficult**	(4) Not difficult if proper regime is followed during pregnancy
**The difficulty of weight management during pregnancy depends on the individual**	(5) Perceptions of difficulty vary from person to person, with influencing factors such as sources of motivation (e.g. focus placed on the baby’s health)

### Weight management during pregnancy is difficult

Some participants reported that weight management during pregnancy is difficult. Common reasons cited for the perceived high level of difficulty are the result of misconceptions on weight management during pregnancy, such as the implications of maternal overweight and obesity during pregnancy and the issues of weight management such as exercise on the fetus.

“I think that managing weight during pregnancy will be very difficult. Pregnant women cannot exercise due to fear of miscarriage. If the pregnant lady eats less during pregnancy, the baby could be affected. The Chinese believes that “one person eats, two people are nourished”. If the pregnant lady is hungry, it could mean that the baby is hungry, so not eating will affect the baby. There could also be gastric pain if the lady eats less. ” (P1)

“It is difficult, because of their mindset. They (pregnant women) may think that being overweight is good for the child.” (P5)

Such beliefs are problematic and can lead to an increased perceived difficulty of weight management during pregnancy. For instance, misconceptions on maternal overweight and obesity during pregnancy that are not corrected can influence a woman’s perception on weight management during pregnancy, leading her to believe that managing her weight is inherently difficult as attempts may have adverse effects on her baby.

Another reason for the difficulty is that the pregnant woman’s family may ‘pamper’ her with foods, and this may inadvertently impede her goal of weight management during pregnancy.

‘With more discipline, it can probably be done. If not, if they have a pampering family where they just keep feeding her, she may think it is OK to eat, since she is pregnant now, unless someone advises her.’ (P13)

Therefore, proper advise must be directed to both the patient and her family, as the family plays a crucial role in weight management of the pregnant woman as well.

Another common reason for participants believing weight management during pregnancy to be difficult was the phenomenon of pregnant women experiencing food cravings and their inability to manage these cravings.

“Of course it is difficult! You think of food in your mind and feel hungry and have appetite. ” (P4)

“I think it is difficult, because they have cravings, and I know it is a psychological thing, but when you really have it, you just can’t push it aside, I don’t know why, it is just ridiculous…. For my last pregnancy I was craving for hard boiled eggs, and I could finish 5 hard boiled eggs at one sitting. It is harder on pregnant women to manage their weight. ” (P15)

These responses suggest that providing overweight or obese women with access to professional guidance on recommended diet regimens during pregnancy and ways to manage their cravings could be relevant.

### Weight management during pregnancy is not difficult

Several other participants believed that weight management during pregnancy is not difficult, and can be achieved based on their experiences. For example, this could be done by following a meal plan during pregnancy or improving their lifestyle based on lessons learnt from previous pregnancy experiences.

“No I don’t think it is difficult. If you are taking healthy food, breakfast with bread or oats, lunch with appropriate size and dinner before 7pm, but take some milk before going to bed.” (P6)

“I don’t think it is difficult, because there was a lot of difference between my first (gained much more weight) and second pregnancy (gained less weight).” (P10)

This suggests that some participants believe that weight management during pregnancy can be done, and more efforts are required to convince women to reconsider their perceived difficulty of weight management during pregnancy. Examples include learning from others who perceive weight management as an achievable task during pregnancy, and have done it before when equipped with the right knowledge.

### The difficulty of weight management during pregnancy depends on the individual

Some participants believed that the difficulty of weight management during pregnancy depends on the individual, for example, in terms of the determination to manage their diet during pregnancy for the baby’s sake.

“I should say that it can be done, but it depends on the willpower of the pregnant lady. Being pregnant, the craving is there, so to ask her to cut down the portion would be quite difficult. However, if the target is on the baby, the mother may think twice and reduce the portion of food consumed.” (P8)

“It depends on the individual. ” (P12)

Their responses suggest that the motivation and will power of the woman plays a role in determining their success in managing weight during pregnancy.

### Perceptions on the importance of weight management during pregnancy (Table 6)

A number of participants believed that weight management during pregnancy is important, however, one participant was unsure, and stated that it was a little important. The most commonly cited reason for this belief was that weight management during pregnancy has positive impacts on both the woman and the baby’s health. This shows that some of the participants have a basic knowledge on the importance of weight management during pregnancy and the implications of material overweight and obesity on both woman and child.

**Table 6.** Perceptions on importance of weight management during pregnancy.

Perception	Sub-themes
**Weight management during pregnancy is important**	Important to/for: Avoid complications to the mother and child during pregnancy and delivery Prevent post-partum weight retention, for the mother’s health Avoid adverse psychiatric issues such as depression after pregnancy, if post-partum weight retention occurs Aesthetic appearance of the mother
**Weight management during pregnancy is a little important**	(5) Unsure of the importance of weight management during pregnancy

Some participants believed that weight management during pregnancy is important. The most commonly cited reason for this belief was that it has positive impacts on the woman and the baby’s health. However, prevention of post-partum weight retention, avoidance of psychiatric issues and aesthetic reasons were also cited as factors which influenced participants to perceive weight management during pregnancy as important.

### Avoid complications to the woman and child during pregnancy and delivery

Several participants believed that weight management during pregnancy is important to avoid complications to the woman and child, which is needed for their safety. They believed that health issues can be prevented if weight is properly managed during pregnancy.

“Yes I do think it is important. This can help to prevent complications for the mummy and the baby, if health issues arise.” (P8)

“I know that it is important, to make sure both the mother and baby is safe.” (P9)

### Prevent post-partum weight retention

Another commonly cited reason was that unsuccessful weight management during pregnancy leads to difficulty losing weight post-pregnancy, which has health implications to the woman. Some participants perceived that managing weight well during pregnancy can help to prevent weight retention post-partum and in turn have better outcomes for the mother.

“It is important for health reasons. Otherwise, you can still have fats after the pregnancy and it is difficult to lose weight.” (P4)

### Avoid adverse psychiatric issues such as depression after pregnancy

Proper weight management during pregnancy was also perceived as helpful to avoid adverse psychiatric issues such as depression after pregnancy, if post-partum weight retention occurs.

“I am sure for a lot of ladies who were pregnant and put on so much weight, after delivery they have difficulty losing it. Some may even be depressed about it. ” (P13)

### Aesthetic appearance of the woman

To some participants, post-partum weight retention also raises aesthetic concerns. Hence, weight management during pregnancy is important so that women can look good after giving birth. Beauty is therefore taken into consideration as a factor in terms of weight management during pregnancy by several of the participants.

“I think mothers are very vain, we want to lose our baby weight. ” (P15)

### Unsure of the importance of weight management during pregnancy

However, the degree of importance of weight management during pregnancy remained ambiguous for one participant. From her perspective, it is a little important, but she is unsure of whether her viewpoint is correct.

‘I am not sure. I think it is a bit important. Basically, it is for the health for the baby’s sake.’ (P3)

Notably, knowledge on the importance of weight management during pregnancy does not necessarily correlate to the ability of participants to successfully manage their weight during pregnancy. While many participants were able to cite that weight management during pregnancy is important, they also pointed out that weight management during pregnancy remains a challenge for women like them.

“Ultimately yes it is very important, but it is also very challenging, because if she didn’t want to lose her weight back then, maybe the motivation will be for the baby. But now that she is pregnant, there is only so much that she can do to move, because in some pregnancies the mummy needs bed rest, or can’t do vigorous exercise, so that is the challenge. ” (P2)

“I think it is important but it is very hard to control. Overweight and obese can lead to effects on blood pressure, cholesterol level and lifestyle. When you are pregnant you are expanding, and you are already breathless when you walk, especially third trimester, so if you are overweight there may be complications. ” (P14)

While knowledge on the importance of weight management during pregnancy may be the first step to changing attitudes of overweight or obese women, knowledge alone may be insufficient and should be supplemented with further specific guidance on to enable these women to manage weight successfully and safely in their pregnancy.

### Levels of motivation for weight management during pregnancy (Table 7)

Conflicting data was obtained on the levels of motivation for weight management during pregnancy. Some women were not motivated on weight management during pregnancy, while others were motivated. Reasons cited include a lack of understanding on the association on the health of the baby and the woman, a sense of security from normal investigation results e.g. of antenatal scans and oral glucose tolerance test (OGTT) for diabetes, the belief that weight management during pregnancy was unnecessary, and pregnancy as a time for enjoyment and indulgence. For women who were motivated to manage weight during pregnancy, reasons were the health benefits to the woman and child, and aesthetic reasons for the woman.

**Table 7.** Perceptions on levels of motivation for weight management during pregnancy.

Perception	Sub-themes
**Not motivated for weight management during pregnancy**	Not motivated, due to/because: Concerns on the baby’s health over the mother’s own health, without realizing the association between the mother’s health and baby’s health Sense of safety when antenatal scans showed that the baby was normal Sense of safety when the mother did not develop gestational diabetes Weight management during pregnancy was deemed unnecessary, as maternal overweight and obesity was thought to have no negative impacts Pregnancy is perceived as the time for indulgence and luxury, and that the pregnancy masks the mother’s overweight status and reduces her guilt from overeating
**Motivated for weight management during pregnancy**	Motivated, due to/because: (6) Health benefits to the mother and child (7) Aesthetic reasons to the mother

### Concerns on the baby’s health over the woman’s own health

Many participants did not feel motivated to manage their weight during pregnancy. They stated that they were interested in the baby’s health and not their weight, but did not realize that maternal weight in fact has implications on the baby’s health, including that of having congenital abnormalities.

“I was not interested in weight management. I was concerned about the baby’s health and that the baby had no abnormalities, and was not concerned about my weight.” (P1)

“During pregnancy, no, not at all, never thought of it (managing weight) and never come across it. I just wanted myself and my baby to be healthy that’s it.” (P9)

### Sense of safety for the baby from normal antenatal scans

Some participants reported a lack of motivation when a sense of safety was felt because antenatal scans were normal. For example, normal growth and lack of observable issues in the baby during check-ups led participants to think that the baby’s health was not compromised.

“There wasn’t any motivation, my motivation was just to see my baby grow healthy. So far during the monthly checkup, baby was growing fine, everything was in order, and I feel fit to go to work, I wasn’t lethargic, only until towards the third trimester, backache started coming in and there was pain at pelvic area, but that’s about it.” (P2)

Interestingly, the above participant’s (P2) third child had atrial septal defect at birth. However, this anomaly was not picked up during her antenatal scans, and even after the birth of her baby, she did not realize that it could be attributed to her pregnancy weight. This suggests that overweight or obese women may lack specific knowledge on how maternal overweight and obesity can cause complications for both woman and child even when the baby has no abnormalities during a particular stage of development, which does not preclude the woman and child from potential complications in the future arising from maternal overweight and obesity such as maternal hypertension and LGA babies.

### Sense of safety on the woman’s health when gestational diabetes did not occur

Also, a sense of security was felt when the woman was found not to have developed gestational diabetes during the pregnancy, which was an outcome that participants were concerned about during pregnancy.

“I ate everything…. there was no limit to what I ate. I ate six to seven times a day. I ate two plates per meal. Two cups of ice milo, watermelon juice, coconut, soy bean drinks etc. Even when they said there could be diabetes, I thought I wouldn’t get it…. And in the end, I really didn’t get diabetes.” (P4)

‘I wasn’t worried about it at all, I didn’t think it was a problem. They said my OGTT was normal, so I continued eating. I was worried about diabetes, and because I didn’t have it, I ate whatever I wanted.’ (P13)

This suggests that it is crucial to inform women that gestational diabetes is not the only or most severe complication that can be threatening to a pregnancy, as many other adverse outcomes may affect the woman and child if an overweight or obese woman does not manage her weight during pregnancy.

### Weight management during pregnancy deemed unnecessary

Still, even more worrying were responses which suggest that a lack of motivation was due to a belief that there is no need to manage weight during pregnancy, as maternal overweight and obesity has no negative impacts on health. Such a belief is dangerous to woman and child and points to the need to educate the public on the implications of maternal overweight and obesity.

“I was not motivated as I just feel happy with my weight and didn’t feel a need to manage weight.” (P4)

“I did not think of it, because I didn’t think it is a big thing at that time.” (P5)

### Pregnancy is the time for indulgence and luxury, masking the woman’s overweight status

Noteworthy was the response of P14, which suggests that some overweight women may believe that pregnancy is the time for indulgence and luxury, and be even less concerned about their weight during pregnancy as their pregnancy masks the fact that they are overweight and reduces the guilt that comes from overeating.

‘During pregnancy I didn’t feel like doing anything, because you want to enjoy the pregnancy, probably after the pregnancy you may think of doing something about your weight. Pregnancy is the only time you can indulge, as you keep expanding and you wouldn’t know, and you wouldn’t feel guilty about eating, and doing what you want as you are pregnant. Maybe after your baby is out you may want to do something about it. Pregnancy is the only time you can enjoy, and eat two portion for the baby, this is the normal concept, as you are expanding you wouldn’t care. Even if you are obese, you think, never mind, I’m pregnant.’ (P14)

While we must take care not to generalize this single response to all overweight pregnant women, it presents a worrying situation as pregnancy is precisely the time when overweight women need to manage their weight as it could have implications on the woman and child.

Several participants interviewed felt motivated to manage their weight during pregnancy. Their main reasons behind this motivation to manage their weight were health benefits to both woman and child, aesthetic reasons, and to lose pregnancy weight.

### Health benefits to the woman and child

Participants who believed that maintaining a healthy weight during pregnancy would be good for both the woman and the baby, reported that it was a factor which motivated them to monitor their weight.

“I was motivated to manage weight during pregnancy, for both myself and my child.” (P6)

### Aesthetic reasons to the woman

Also cited a factor affecting the perceived importance of maintaining a healthy weight during pregnancy, and several participants were motivated to manage their weight to look aesthetically appealing. These participants believed that regaining their usual shape after pregnancy would be easier if excessive weight gain had not occurred during pregnancy.

“I felt motivated to manage my weight so that I would slim down faster after giving birth.” (P12)

These motivations are also important reasons which spur overweight and obese women to manage weight during pregnancy. Healthcare professionals may keep these in mind when providing patient education and counseling to overweight and obese pregnant women, to give them a broader perspective on ‘push factors’ to manage their weight and remind them of the other reasons they may wish to manage their weight.

### Experiences of women on weight management during pregnancy (Table 8)

Several participants indicated that they tried to manage their weight during pregnancy. This is not promising, as even among participants who had knowledge that weight management during pregnancy is important, this awareness did not always translate to action.

**Table 8.** Experiences of women on weight management during pregnancy.

Experience	Sub-themes
**Tried ways to manage weight during pregnancy**	A minority of participants tried to manage weight during pregnancy through methods such as diet or exercise. This is done by: Learning from past experiences (i.e. mistakes made in first pregnancy) Seeking resources through family members who may know about weight management during pregnancy Online platforms such as Facebook
**Improvements to be made on experiences of weight management during pregnancy**	(4) Weight management during pregnancy can be done safely and effectively during pregnancy through professional guidance e.g. from gynecologists and dietitians (5) Guidance from professionals is preferable to selecting a regime on one’s own without reliable information (6) Professional help is especially important when women have little to no knowledge on weight management or have misconceptions regarding the subject

### Learning from past pregnancy experience

One participant mentioned that she learnt from her past pregnancy experience and avoided the mistakes of eating anything she wanted during her pregnancy, and not limiting her food intake. In her second pregnancy, she was more careful to avoid overeating, and to make wiser food choices.

‘I learnt through mistakes…. For my second pregnancy I was motivated…. Eat healthy food, sufficient food for you and the child, don’t overeat.’ (P10)

However, learning from past pregnancy experiences would mean that women would have to make at least one mistake in terms of weight management during pregnancy, before she gains insight and experience to manage her future pregnancies better. Therefore, although this way of learning may be effective, it is not recommended as a preventive approach.

### Learning to manage weight through family members

Other methods include seeking help from immediate family members. In the case of a participant (P15), her husband had contacts with dietitian and allied health staff, hence she likely had access to better information in comparison to the general population.

‘(I managed my weight during pregnancy) through exercise and eating well. I haven’t seen a dietitian or allied health staff, but I go to my husband (a fitness trainer) for help, and he knows some dietitians and nurses from hospital. It was not through referrals.’ (P15)

Although learning to manage weight through family members is useful for some, it may have poor outcomes if family members provide advice which are misinformed. Furthermore, the above participant’s husband was especially well-connected with people who likely have ample resources in the subject, such as dietitians and nurses. Indeed, these resources may not be accessible to other women who are less well-connected.

### Learning from online platforms

One participant (P10) indicated that she looked for and participated in programs or support groups on weight management during pregnancy on her own accord. Thereafter, she joined online groups to know more about the experiences of other women, and felt that support groups can be a good way to educate women on the implications of maternal overweight and obesity and motivate them to manage their weight.

“I heard some things about that, especially on Facebook, such as those mother groups, where a lot of them still say, no weight management or diet during pregnancy, do whatever you want to do, eat whatever you want to eat, just be normal, but don’t be too overweight. These are closed groups like “We are pregnant”, “Mummies and babies”. (P10)

Although the use of such platforms may be helpful to some, they must be used with caution as the advice they disseminate may not be medically appropriate, and may not apply to women who are considered to have high-risk pregnancies. For instance, some groups that the participant came across encouraged women not to manage their weight during pregnancy.

### The dangers of online platforms

Furthermore, the moderators of these groups are unknown. It appears that these groups may still appeal to women who wish to know more about topics on pregnancy, even though the ideas shared are from strangers. It is uncertain if pregnant women heed the advice of these online platforms. More efforts should be done to check the accuracy of the information provided on these platforms.

“I have no idea who are the moderators of these groups actually, I just joined when I saw the pages, as I just wanted to know more about mummies and babies. They are like strangers, but they are all Singaporeans or foreigners who stay in Singapore, who are sharing their lives, their ideas and everything in the group.” (P10)

Well-meaning advice on these online platforms may contain medical errors, or may be ill-tailored for specific groups of pregnant women such as those who are overweight or obese. Online platforms such as social media may be able to reach out to women who are eager to learn and curious about advice for pregnant women in the community. It may be helpful to consider regulating such online platforms, to keep it up-to-date with accurate information.

### Improvements on weight management during pregnancy can be improved: the role of healthcare workers

Many participants mentioned that women can manage their weight safely and effectively during pregnancy through professional guidance from their gynecologists and dieticians, which is preferable to them selecting a diet without the necessary information.

“I think doctors should refer them to a professional like a dietitian, rather than them managing on their own, without knowing what is best for them.” (P8)

“ My gynae didn’t tell me anything, probably if she told me I would have been more worried…. At the very early stages (of pregnancy) when women come to see the doctor, at that time doctor can probably advise them.” (P13)

“I think they (pregnant women) should seek proper advise from their gynae, who can refer them to the right people, I don’t think they should jump blindly on a diet, it is a matter of choosing the right food.” (P15)

Engaging professionals will also be particularly useful for women with no understanding or have preconceived notions on weight management during pregnancy.

“I have no idea how to safely manage weight during pregnancy.” (P1)

“I’m not so sure. I think exercise is not for pregnant women.” (P3)

Many participants reported that they had little understanding on methods on weight management during pregnancy, and would benefit from expert advice in this matter.

## Discussion

Our study found that there are gaps in knowledge, misconceptions, as well as a range of perceptions of overweight and obese women during their pregnancies. Some of these, such as the misconception of the need for ‘eating-for-two’, were similar to previous studies which we will elaborate on below. On the other hand, there was no reported perception on the cost and convenience of food as a determinant in their choice of diet in our study population, which is in contrast to that reported among African-American women in the USA. We have also identified potential determinants in our population, such as marital status, which may play a role in women’s knowledge and perceptions on weight management during pregnancy, which have not been reported by other studies to date.

### Addressing the knowledge gap and misconceptions

A study from the UK [30], found that women’s midwives give thorough information on what not do during pregnancy, but were seldom offered information on what they should do with regard to food intake and physical activity for weight management. In contrast, no mention of midwives was elicited from our participants’ responses, and their information was often obtained from the community or family. Another study [31] conducted in Pennsylvania on postpartum women who were overweight or obese prior to pregnancy, found that many women with excessive gestational weight gain described ‘eating-for-two’, and reported exercising less during pregnancy. The findings of this study are similar to ours, although our participants also cited other misconceptions and perceptions in the Asian community, which could exacerbate the problem of maternal overweight and obesity.

Our findings showed that participants had minimal to no knowledge of the repercussions of maternal overweight and obesity on pregnancy and birth outcomes, and various misconceptions on weight management during pregnancy. The knowledge gap and misconceptions have the potential to alter pregnant women’s decisions on their lifestyle such as diet and exercise. If uncorrected, these perceptions could potentially lead to adverse health effects for both the woman and child, and lead to dangerous pregnancies. Misconceptions can be largely attributed to false information on the internet, passing down of family’s, relatives’, or friends’ beliefs in old wives’ tales, as well as their false correlations between lifestyle and pregnancy outcomes. There is a pressing need to directly address these beliefs with patients who will be likely affected by these.

Potential solutions to close this knowledge gap and correct these misconceptions so as to minimize barriers and promote motivation for diet and exercise in overweight and obese pregnant women should focus on educating and building awareness, targeting both the woman and their family members and friends.

### A need for international consensus

Currently, maternal weight is a concern all over the world. Yet, there is a lack of international consensus on the content of guidelines, recommendations or interventions which are most efficacious with respect to maternal weight, and how health policies impact clinical practices and outcomes for the woman and child [37]. We would like to call for guidelines from international organizations such as World Health Organization or FIGO (International Federation of Gynecology and Obstetrics) to release guidelines on what can be done for overweight and obese women to improve maternal and child health, so that nations can modify and adopt these guidelines to suit their specific cultural and socio-economic context.

We also encourage future research to test for the safety, effectiveness and applicability of current and new strategies for overweight and obese women during pregnancy. A recent finding by Chang et al. [38] showed that the water ought to be better studied in the research of weight management and clinical strategies. An essential nutrient in population’s diet, potable water is usually an inexpensive commodity that is often widely available and accessible by people. Furthermore, in comparison to health services such as special diet and exercise plans for pregnant women, encouraging fluid intake in women who have difficulty managing their cravings during pregnancy or eat excessively for other reasons may play an important role for overweight and obese pregnant women. However, its safety and effectiveness for women in the pre-conception, child-bearing and post-partum period on maternal and child health is unknown. More studies can be conducted on the effectiveness of increased hydration among overweight and obese pregnant women.

### Developing culturally specific clinical pathways

According to Reyes et al [32], low-income, overweight, African-American women tend to choose foods that had high levels of fats and sugars due to its taste, cost, and convenience. In addition, women had misunderstandings on the definition of healthy, e.g. believing that ‘juice is good for baby’, which led to overconsumption. In our study population, no participant cited cost and convenience as factors when choosing their food for consumption. However, in other Asian regions with different economic contexts, food prices and accessibility may play a part in their choice of diet. Therefore, different regions should modify patient care pathways according to their cultures and socio-economic statuses.

An Australian study [33] found that maternal education status is a main determinant of the extent of knowledge. Although we were unable to accurately determine the influence of education on participants’ extent of knowledge, we found that even participants who had demonstrated fair knowledge on the problems of maternal overweight and obesity, reported difficulty managing their weight during pregnancy. Therefore, on top of educational campaigns, we believe that equipping overweight and obese women with resources and enabling factors such as support from professionals in nutrition and exercise, and committing them to a clinical pathway, is needed for effective weight management during pregnancy.

We also found that marital status could be a potential determinant of the extent of knowledge and motivation of participants in weight management during pregnancy, for example, from the influence of their spouses on their choices and actions. For instance, one of our study participants reported that she exercised during her pregnancy as her husband was a fitness instructor and could advise her on exercises which she can do. However, to our knowledge, marital status and spousal influence has not been reported as a determinant in previous studies, even though it was found in our data to be a potential factor. We would encourage future studies to focus on this factor to determine its weight as a determinant, and to explore how it affects the choices of women, whether in terms of diet, exercise or other choices during their pregnancy.

### Study strengths and limitations

A strength of our study is that we conducted one-to-one interviews in a private setting, such as an unoccupied clinic consultation room, where the participants may be more likely to give genuine responses as opposed to a focused group discussion (FGD) which could have limited the confidentiality and anonymity of participants and their responses as they interact and listen to one another.

We acknowledge that this study has small sample size with only 15 participants which can undermine its credibility [39]. However, no computations can be done in qualitative studies to ascertain the minimum number and kinds of sampling units required [40], and this limitation may be compensated for by the quality of input from the participants as the quality of information obtained per sampling unit, as opposed to their number, plays a more important role [40]. Furthermore, small is considered to be beautiful where sampling in qualitative research is concerned [41]. In terms of reliability, the process of qualitative studies is under-standardized and relies on the insights and ability of the observer, hence the trustworthiness of the findings is weakened [34]. However, we performed member checking to give input on our data and to provide a balanced viewpoint on the adequacy and accuracy of our analyses. We took the perception of reality from an interpretivist point of view and aimed to depict the truth as accurately as we possibly could.

## Conclusions

In our study, we have identified the levels of knowledge, misconceptions and other factors that play a role in weight management during pregnancy for overweight and obese women. For example, we found that levels of knowledge are diverse, ranging from no knowledge to correct and specific examples on implications to the mother and child, as well as identified misconceptions, such as a need to consume twice the usual portion of food during pregnancy and hunger from the mother is due to hunger from the baby, and established other influencing factors such as ‘pampering’ of the expectant mother during pregnant which is cited as a reason for the perception of difficulty in weight management during pregnancy.

These findings may be used when guidelines are tailored for Asian regions, tweaked to suit their socio-economic status. The development of national and international clinical pathways for overweight and obese women is warranted, so that women have awareness of the implications of overweight and obesity during pregnancy, and are empowered with resources to manage their weight safely and effectively.

## Paper context

Overweight and obesity among pregnant women is associated with adverse reproductive health outcomes. Perceptions of women during pregnancy, ranging from White, Latina and African-American women from the West are well-studied. Given that socio-cultural differences between Asian and Western populations exist, this study is the first to add an Asian perspective to the discussion, delineating their beliefs and attitudes, and highlighting a need for more strategies and guidelines that are specific to cultural and socio-economic contexts.
